# Atmospheric ammonia (NH_3_) emanations from Lake Natron’s saline mudflats

**DOI:** 10.1038/s41598-019-39935-3

**Published:** 2019-03-14

**Authors:** L. Clarisse, M. Van Damme, W. Gardner, P.-F. Coheur, C. Clerbaux, S. Whitburn, J. Hadji-Lazaro, D. Hurtmans

**Affiliations:** 1 Université libre de Bruxelles (ULB), Atmospheric Spectroscopy, Service de Chimie Quantique et Photophysique, Brussels, Belgium; 2 The University of Texas at Austin, Marine Science Institute, 750 Channel View Drive, Port Aransas, Texas 78373 United States; 3 LATMOS/IPSL, Sorbonne Universités, UVSQ, CNRS, Paris, France

## Abstract

In a recent global analysis of satellite-derived atmospheric NH_3_ data, a hotspot was observed in the vicinity of Lake Natron, Tanzania. The lake is in the centre of an endorheic (limited drainage) basin and has shallow, saline-alkaline waters. Its remote location and the absence of nearby large anthropogenic sources suggest that the observed NH_3_ is mainly of natural origin. Here we explore 10 years of IASI NH_3_ satellite data and other publicly available datasets over the area to characterize the natural NH_3_ emissions in this unique ecosystem. Temporal analysis reveals that the emissions are episodic and linked with the lake’s surface area. The largest NH_3_ column loadings generally occur at the end of the dry season in September–November over Lake Natron’s largest mudflat, that is exposed with receding water levels. The timing is different from the agricultural dominated NH_3_ emissions in the wider Natron area, which peak early in the year, after the first wet season. The likely source of NH_3_ at Lake Natron is decomposition of organic material, either from rivers and springs or produced in the lake (plankton, bird excreta). High temperatures and alkalinity are known to promote NH_3_ losses from soda lakes. We formulate six processes that may explain why the largest losses are observed specifically over concentrated brines and/or exposed sediments. As a by-product, we also show that hyperspectral infrared sounders such as IASI are capable of mapping different types of evaporative minerals such as trona and thermonatrite.

## Introduction

Ammonia (NH_3_) plays a critical role in the global biogeochemical cycle of nitrogen^1^ as one of the key components of reactive nitrogen. Largely due to the widespread availability of industrially fixed nitrogen^2^, atmospheric emissions of NH_3_ are increasing steadily^3,4^, with devastating effects on air quality, ecosystems and climate^5^. Sources of atmospheric ammonia include animal waste, fertilizers, combustion (biomass burning, waste burning, transport), industry (production of chemicals, manufacturing processes), soils, plants and oceans^3,6,7^.

About a decade ago, it was discovered that infrared satellites can detect and measure atmospheric NH_3_, which resulted in the first measurement-based global maps of its distribution^8^. Satellite measurements are currently available from four instruments: AIRS^9^, TES^10^, CrIS^11^ and IASI^12^. Recently^13^, a hyperresolved (0.01° × 0.01°) world map of NH_3_ was presented, following the combined exploitation of all available IASI satellite data over ten years and a series of algorithmic improvements^14^. Careful analysis of this map revealed 248 NH_3_ emission hotspots. About one third of these hotspots were attributed to high-density animal farming, but surprisingly, the majority were linked to industrial activity, in particular to chemical fertilizer production plants.

**Figure 1:**
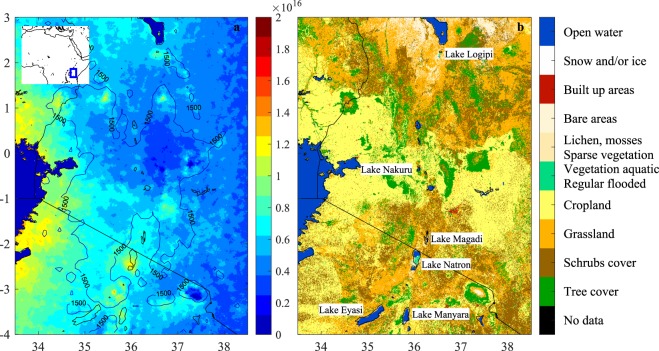
Eastern African Rift and surrounding area in Tanzania and Kenya (the location within Africa is shown in the inset). (Panel (a)) NH_3_ column loadings (molec/cm^2^) averaged from 2008–2017 IASI data. The IASI average here, and elsewhere in this paper were calculated using an oversampling method^13^. NH_3_ column loadings over the largest water bodies were set to zero (Lake Victoria in the West and Lake Turkana in the North). Also shown in the left panel is the 1500 meter altitude contour line. Lake delineations are from the GSHHG-‘fine’ dataset^92^. (Panel (b)) Land cover data based on Sentinel-2A observations from December 2015 to December 2016.

**Figure 2:**
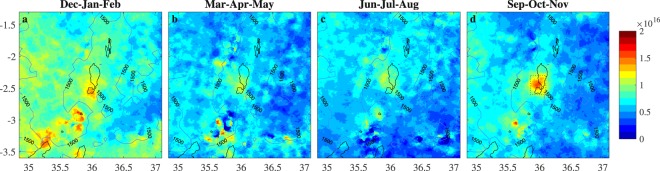
Seasonal NH_3_ column loading averages (molec/cm^2^) from IASI measurements onboard Metop A for the period 2008–2017. The corresponding averages for IASI on Metop B are provided in Supplementary Information (Fig. S1). The hotspot area, indicated with a rectangle in panel (d) is used for timeseries analysis (Figs 4 and 5). The entire area shown was used to calculate the background NH_3_ column loadings in those figures.

The hotspot over Lake Natron, Tanzania (Figs 1 and 2) was the only one that was identified as having a natural origin. Yearly averaged emission fluxes were estimated to be of the order of 15 kt/year (with an estimated lower and upper bound of 4 and 180 kt/year). In this paper, we use the 2008–2017 NH_3_ IASI data and other publicly available datasets to help understand the nature of the emissions. In particular, in Sec. 3 we analyse spatial and temporal patterns and link these with available hydrological parameters. We show that the largest NH_3_ column loadings are observed over Natron’s exposed salt encrusted mudflats. This result leads to the question whether the observed NH_3_ enhancements are actually genuine and not due to a surface retrieval artefact. In Sec. 4 we discuss surface emissivity features and provide the necessary spectroscopic evidence. Based on the available data, we discuss in Sec. 5 possible important sources and mechanisms driving the emissions. We start with providing the necessary background information on the lake.

## Lake Natron

**Figure 3:**
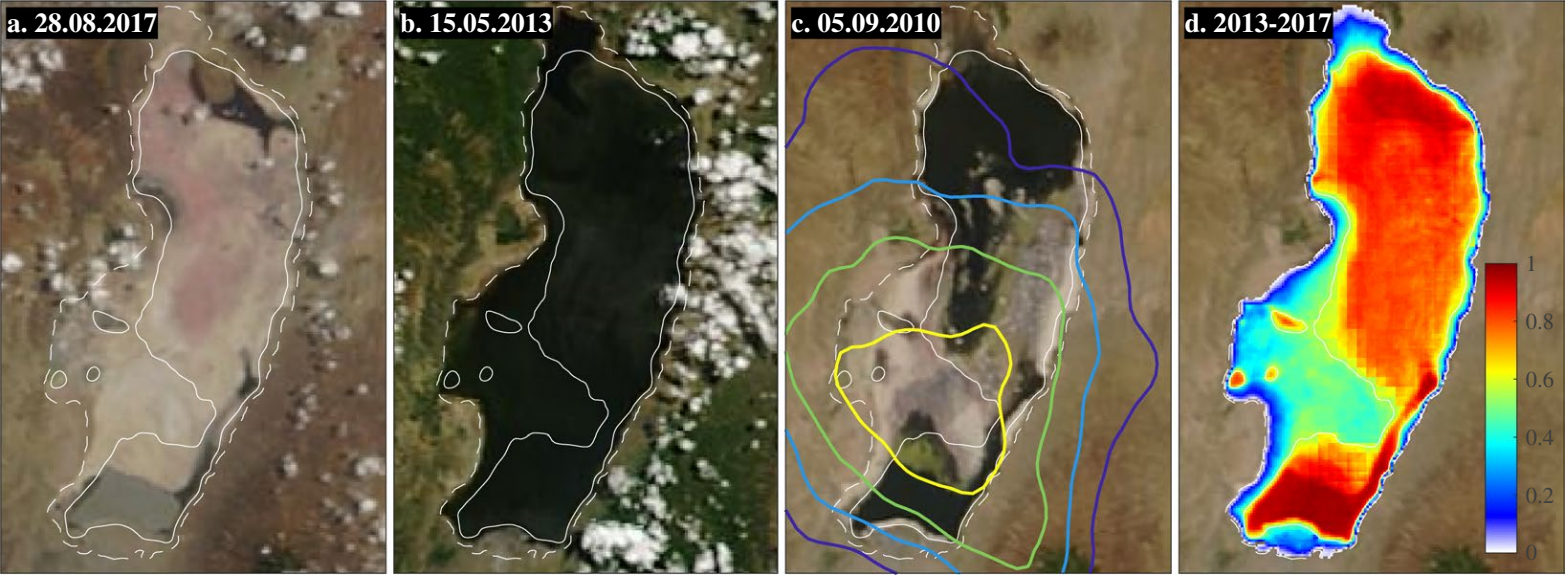
Variations in water extent illustrated and derived from MODIS imagery (MODIS corrected reflectance imagery from NASA Worldview). Panel (a) shows the lake almost completely dry, panel (b) shows the lake at its maximum extent (see also Figs 5 and 6 of ref.^16^). The dates of these two extremes were found using the MODIS water product from the period 2013–2017. The frequency of detected water over each ~250 × 250 m^2^ area, as derived from the MODIS water product, is shown in panel (d). The 0.01 (almost always dry) and 0.55 (flooded more than half of the time) frequency lake contours are indicated on the other panels with white dotted and solid lines, respectively. Panel (c) shows the situation on 5 September 2010, a day for which IASI measured large localized increased NH_3_ column loadings. The 2008–2017 NH_3_ averaged column loadings are shown as coloured contours in panel (c) and visualize the location of the NH_3_ hotspot (the contours correspond to 0.8, 0.9, 1.0 and 1.1 10^16^ molec/cm^2^).

Located in the Arusha region in North Tanzania on the Kenyan border (Fig. 1b), Lake Natron^15–17^ is one of many lakes located in the Eastern Rift Valley, which runs from Tanzania over Kenya and Ethiopia towards the Afar triangle. The lake is at an altitude of ~600 m above sea level surrounded by plains and several volcanic mountains. Its maximal surface area measures about 875 km^2^ (~50 km long and ~20 km wide) and the water depth ranges from a few centimetres to several meters. Water inflow comes from rivers, direct rainfall on the lake’s surface and a large number of (hydrothermal) springs. Lake Natron has no outflow mechanisms other than evaporation, and as the climate is hot and arid, the hydrological balance is generally negative. Periodically, the lake dries out completely, with the exception of a few isolated water bodies (commonly referred to as lagoons) near the inlets of the rivers and springs. Figure 3 shows the lake at different levels of water surface extent.

Lake Natron is an archetype soda lake^18,19^, with very large concentrations and deposits of sodium, carbonate and chlorine, carried in by water from the surrounding rocks and that as a closed basin, accumulated over time. The salinity levels of the lake water (i.e. brine) vary significantly depending on the water level and the proximity of fresh water inlets. The main soda ash (Na_2_CO_3_) deposit is located in the central to north-eastern part of the lake in the form of a vast, 1.5 m thick trona pan^17^. Saline mudflats surround the central salt deposit and the permanent lagoons. These flats get flooded regularly and leave a mud surface behind encrusted with efflorescent salt crystals after drying.

As a consequence of its chemical composition, the lake is highly alkaline with reported pH levels in the range 9–11.5. Yet, despite this uninviting climate, it is extremely productive biologically due to a number of well-adapted plankton (bacteria, archaea, diatoms and green algae) which thrive under Natron’s abundant CO_2_, nutrients, and year-round elevated light intensities and temperatures^18–22^. The most important organisms are cyanobacteria such as arthrospira fusiformis (spirulina). Haloalkaliphilic archaea start to bloom under high salinities and give the lake an orange-pink-red appearance, especially observed in the upper (northern) half of the lake.

As with other lakes in the region, Lake Natron and its surrounding wetlands are important for various waterbird species, in particular flamingos who feed on the abundant phytoplankton. Lake Natron is the main breeding place for the *lesser flamingo* (Phoenicoparrus minor). They build nests on the mudflats or on the main trona pan when conditions are suitable. The remote and toxic environment offers protection from predators^16,23,24^. The lake is also home to a particular fish species (Alcolapia) that survives in the relatively fresh hot-spring waters^25,26^.

The population in the Natron basin is limited to a handful of villages scattered around the lakeshore. Each village has a population of a few 1000^27^. The main landuse and resource for the local population is livestock^28^. Sheep, goats and cattle are herded in a semi-nomadic manner on the grasslands surrounding the lake, the rift escarpment or up into the mountains^27^. Small-scale agriculture takes place on the western lakeshore near the Moinik and the Peniji river mouths^27^. Industrial extraction of natural soda ash from the trona deposits was considered for a long time at Lake Natron^29^, but plans were finally cancelled due to environmental concerns^30,31^. Tourism is very limited^32^ because of the lake’s remote location.

## NH_3_ at Lake Natron: where and when

The IASI-derived 2008–2017 NH_3_ column loading average is shown in Fig. 1a. A close-up of the hotspot is shown in Fig. 3 (panel c) superimposed over visible imagery with the lake at intermediary water levels. Its centre is located near the lower-half of the lake over the mudflat between the main trona pan in the north and the lagoon in the south. It is the largest mudflat of Lake Natron^17,33^ and is sometimes referred to as the Gelai mudflat^23^. As demonstrated in the three figures of the lake at different water levels, the mudflat floods periodically but is also one of the first areas to dry out when water levels recede. The availability of the daily MODIS water product^34^, allowed us to quantify the frequency of flooding at a ~250 × 250 m^2^ resolution for the period 2013–2017 as illustrated in Fig. 3d (0 means always dry, 1 means permanently flooded). Observe the permanent large southern and northern lagoon, as well as smaller lagoons on the west and eastern shoreline. The mudflat area, over which the NH_3_ hotspot is observed, is exposed about 50% of the time. MODIS imagery is the corrected reflectance imagery from NASA Worldview.

Seasonal NH_3_ column loadings averaged over Lake Natron and the immediate surroundings are shown in Fig. 2 for IASI onboard Metop A and in Fig. S1 in the Supplementary Information for IASI onboard Metop B. Both the hotspot and the background area exhibit a strong seasonal cycle. The largest NH_3_ column loadings and sharpest spatial gradients are observed over the hotspot during September to November. The NH_3_ measured in these months evidently determines the location of the hotspot on an annual basis. However, a local NH_3_ maximum is seen close to the lake during all seasons. The hotspot is slightly displaced to the south-east and diluted during the months December to February. The NH_3_ column loadings in the valley floor, and near Lake Victoria are also highest in this season. The period March to August has the lowest column loadings both in the surrounding area and over Lake Natron.

**Figure 4:**
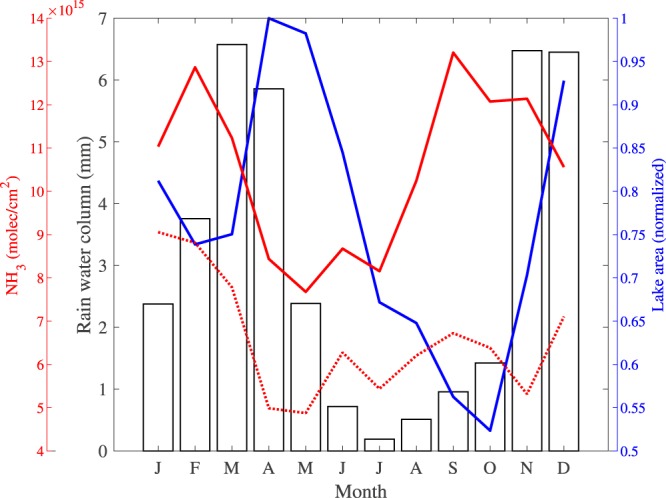
2008–2017 monthly averages of the rainfall amount (histogram, derived from ECMWF’s ERA5 data on the model output at 35.91°E, 2.49°S), the normalized lake area (blue, derived from the MODIS water product), the IASI NH_3_ column loadings (molec/cm^2^) over the hotspot area (solid red line) and over the background area (dotted red line). Note that the lake area follows with some lag the rainfall cycle.

Before continuing investigations of the hotspot, we discuss the observed seasonality of the background area (the entire area shown in Fig. 2). Monthly average NH_3_ column loadings over the background are shown in Fig. 4 (dotted red line). The background column loadings in January-February are almost double those in the other months. Meteorological variables provide clues as to the origin of this seasonality. Temperatures do not vary significantly throughout the year at Lake Natron. However, the lake has two wet seasons (see Fig. 4): a short one in November and December, and a longer one from February to May. A long dry season from May to October separates the two wetter periods. Biomass burning is an important emitter of atmospheric NH_3_^35^, especially in Africa, but the seasonality of the wet seasons immediately excludes fires as the origin of the background NH_3_. The majority of the fires in the area take place in the Serengeti National Park (~100 km west of Lake Natron), in the middle of the dry season, between June and August (as shown in Fig. S2 in the Supplementary Information). Note also that the number of fires in the immediate vicinity of the lake is extremely low. Most of the observed background NH_3_ likely originates from agriculture as the observed NH_3_ average correlates spatially with livestock numbers^36^. In particular, the largest background values of NH_3_ are observed near Lake Victoria where livestock densities are highest (Fig. 1). The seasonality of these emissions relates to the fact that NH_3_ from animal excreta is only volatilized after chemical breakdown (hydrolysis) of urea, which requires sufficient amounts of water^37^. This precipitation effect has also been noted in other dry savannas in Africa^38^. More generally, rainfall stimulates organic matter mineralization in arid areas^39^. On the other hand, heavy rain transports dissolved NH_3_ further down in the surface^40^, which could explain why the largest NH_3_ emissions occur between the two wet seasons, when the soil is presumably at intermediate moisture levels.

**Figure 5:**
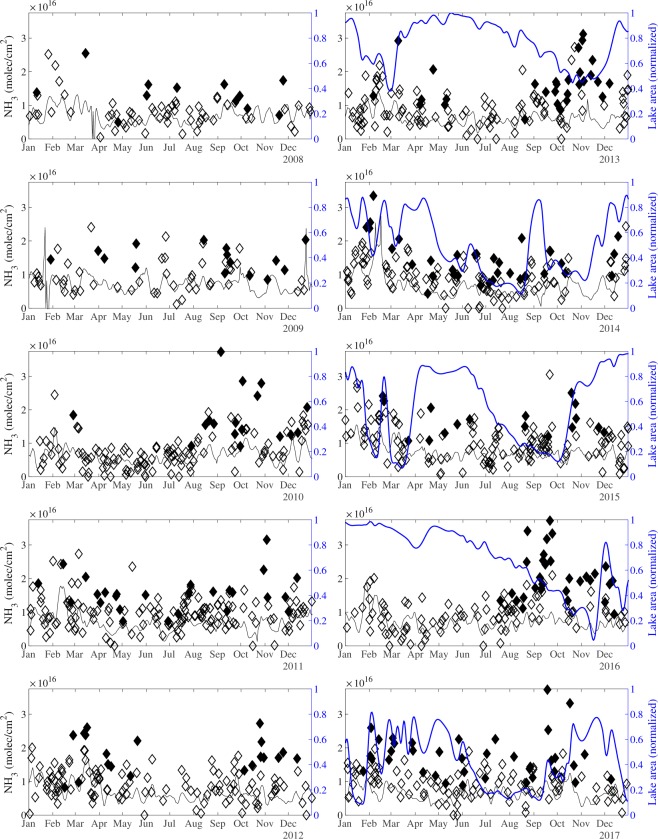
Timeseries of atmospheric NH_3_ (molec/cm^2^) over Lake Natron. Individual NH_3_ column loadings observed over the hotspot area are plotted with diamond symbols. Solid diamonds indicate that the observed NH_3_ column loading over the hotspot area is a local maximum, that is higher than the other measured column loadings of the larger background area. Mean background column loadings, calculated over an area that extends the hotspot area by 1° on all sides (see Fig. 2), are represented with solid black lines. The MODIS water extent, expressed as fraction of the maximum is shown in blue for the period 2013–2017. To simplify analysis, both the background column loadings and the water fraction were smoothed in time.

Returning to the hotspot, the monthly timeseries in Fig. 4 (solid red line) confirms that the largest average column loadings occur from September to November, in addition to the (background) maximum in February. The normalized lake surface area shown in Fig. 4 (blue line) show that these maxima coincide with the end of the dry season in October-November, when the lake area is at its smallest, and between the two wet seasons, with a local minimum in surface area. To explore the link further between NH_3_ column loadings and lake surface area, it is useful to look at shorter time scales. Daily NH_3_ column loadings over the hotspot area are shown in Fig. 5 for the entire period 2008–2017. Symbols are filled when the individual (daily) measured column loading over Lake Natron is the highest of the entire background area. Average background column loadings are indicated with black solid lines. For dates after 2013, the MODIS surface water extent is also shown. Note the large interannual variability of both NH_3_ and water extent. In certain years, strong emissions occur throughout the year (e.g. in 2011, 2014 and 2017). In other years, atmospheric NH_3_ emissions are episodic and separated at times by long quiescent periods. Such episodes took place e.g. in August–October 2010, September–November 2013 and August–November 2016. Shorter flares of the order of a week occur during October–November in most years. The variations in NH_3_ relate closely to the water extent, as seen for example for the prominent NH_3_ episodes in 2013 and 2016 that coincide with receding water levels. Prior to these episodes there was a long period of high water and NH_3_ column loadings that rarely exceeded background levels. On the other hand, throughout 2014 and 2017 large column loadings were observed and with a water fraction below 0.8, the lake never reached its maximum extent in those years. From this analysis we conclude that the largest NH_3_ emissions occur with the drying of Lake Natron’s mudflats.

Before exploring possible mechanisms to explain these emissions, it is important to exclude other possible processes that could explain the observed seasonality. Agricultural emissions from the surrounding areas, and pastoralism in the Natron basin may contribute to the peak in the wet season. However, herding is too disperse around Lake Natron to explain the intensity or the location of the hotspot directly over the lake’s mudflat, where livestock never comes. The different seasonality and limited spatial extent of the hotspot indicate that another mechanism drives the majority of the hotspot’s emissions. A potential explanation of the observed seasonality could be found in the existence of possible seasonal biases in the NH_3_ measurements, related in particular to variations in thermal contrast and the planetary boundary layer (PBL) height. Thermal contrast (which is the difference between the temperature of the surface and air) is known to largely affect measurement sensitivity in the infrared spectral domain^41^. However, the Lake Natron area provides almost ideal conditions for accurate and sensitive satellite measurements of NH_3_ with a stable mean thermal contrast of ~10 K for all months at the overpass time of IASI (9.30 in the morning). The PBL height affects greatly the vertical distribution of NH_3_ and as the NH_3_ retrieval algorithm assumes a fixed constant vertical profile, large seasonal differences in the PBL height could result in a seasonal biases in the retrieved columns (ref.^42^ reports biases of around 50% between a PBL of 100 m versus that of a 2 km one). An analyses of ERA5^43^ PBL heights at Lake Natron however, shows very little seasonal variation (at the IASI overpass time the yearly average is 984 m with a standard deviation of 253 m, a minimum monthly average of 866 m in June and a maximum of 1119 m in October). Another aspect that should be considered is that of wind dispersion. NH_3_ total column loadings that satellites measure only correlate well with emissions if the NH_3_ atmospheric lifetime is constant. For instance, strong winds in one season could reduce the build-up of NH_3_ directly above the lake. ERA5 surface wind fields indicate a marked seasonality in wind speed strength, with the strongest winds (2.2–2.8 m/s) in the period May to October, and weaker winds (1.7–2 m/s) in the other months. If the lack of dispersion would explain the seasonality, a NH_3_ maximum could not be seen in September. Wind directions appear randomly distributed, with no apparent seasonality. Finally, we consider and exclude the existence of measurement artefacts, which is the subject of next section.

## Spectroscopic evidence

Since no obvious NH_3_ source was initially identified at Lake Natron, it was early on suspected that the hotspot was due to a retrieval artefact rather than an actual NH_3_ enhancement. Retrieval artefacts may occur when spectra exhibit unusual features in the spectral region of interest. These ‘unusual features’ are due to atmospheric constituents that are difficult to model (e.g. aerosols^44^) or specific surface types (e.g. sand, snow and ice^45^).

**Figure 6:**
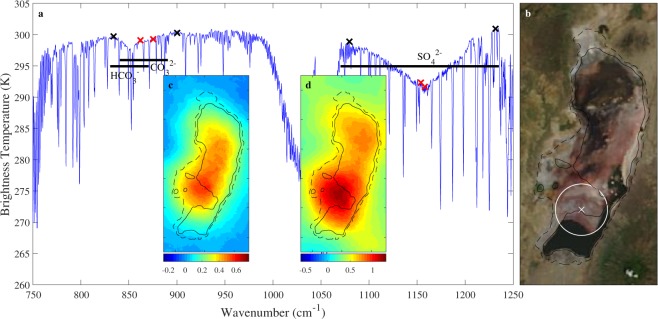
IASI spectrum observed over Lake Natron on 15 December 2007, evening overpass is shown in panel (a). Only the part of the spectra most relevant for the NH_3_ retrieval is depicted. The location of the IASI footprint is shown in panel (b) on the right superimposed over visible MODIS Terra imagery from the day after (the MODIS image of the 15th is more blurry but similar). Panel (a) also shows the position of the three large identified emissivity features, as indicated by thick black lines and their suspected associated functional groups. Mean brightness temperature differences between two baseline channels (black crosses) and two affected channels (red crosses) quantize the emissivity features. Selected channels are in increasing order of wavenumber: 833.5, 861.5, 874.5, 899.75, 1079.25, 1153.75, 1157.75 and 1231.5 cm^−1^. Since the two features near 850 cm^−1^ partially overlap, they were characterized by a single brightness temperature difference. The insets c and d show the 10-year oversampled average of both brightness temperature differences calculated using all 2008–2017 IASI observations (morning overpass). MODIS imagery is the corrected reflectance imagery from NASA Worldview.

Surface effects manifest themselves especially in the ‘atmospheric window’ between 800–1200 cm^−1^. This spectral range is largely transparent for infrared radiation, so that the largest portion of the radiation observed on top of the atmosphere originates from the surface. Hence, spectral variations in surface emissivity, expressing the deviation of the surface from the theoretical black body, impact the observed spectrum^46^. The peculiar visible appearance of Lake Natron with its salt encrusted (mud)flats, and the fact that the NH_3_ retrieval relies fully on the atmospheric window, therefore calls for a careful investigation of possible surface retrieval artefacts. An earlier analysis of outliers in the principle component reconstruction of IASI spectra, revealed that soda lakes can indeed exhibit very large and sharp emissivity features^47,48^. The most extreme example for Lake Natron, for the entire period of IASI observations, is shown in Fig. 6. It was observed on 15 December 2007 right above the main mudflats. Although this example does not reflect common observations over Lake Natron, it helps identifying the relevant features, and illustrates the worst case behaviour.

A large feature between 1070 and 1240 cm^−1^ is apparent as well as a smaller one between 830 and 890 cm^−1^. The larger one may relate to a mineral with a ${{\rm{SO}}}_{4}^{2-}$ functional group, which has a strong vibrational mode in this range^49,50^. There is a good match with the emissivity spectrum of gypsum, but this mineral is highly unlikely to be seen over Lake Natron given its alkaline Calcium-deprived soil^51^. A plausible candidate is kogarkoite [Na_3_(SO_4_)F], previously identified at Lake Natron^17,52–54^. Its transmittance spectrum^55^ is compatible with the observed feature. Other candidate sulfate minerals include thenardite [Na_2_SO_4_], burkeite [Na_2_CO_3_ · 2Na_2_SO_4_] and mirabilite [Na_2_SO_4_ · 10H_2_O], but these have to our knowledge not been identified at Lake Natron.

The dip in the spectrum between 830 and 890 cm^−1^ is composed of two features, one centred around 853 cm^−1^ and a broader one between 840 and 890 cm^−1^. This is not apparent from this example spectra, but a comparison with other spectra reveals that both features occur with varying intensity. The feature around 853 cm^−1^ may relate to vibrations of the ${{\rm{HCO}}}_{3}^{-}$ group as it matches nicely with published reflectance spectra of trona (Na_2_CO_3_ · NaHCO_3_ · 2H_2_O)^56,57^. The broader feature is likely due to the ${{\rm{CO}}}_{3}^{2-}$ group^49^. Natron (Na_2_CO_3_ · 10H_2_O) and thermonatrite (Na_2_CO_3_ · H_2_O) have spectra that are compatible (see^56^ and the RELAB spectral database). However, natron can be excluded as it does not precipitate at Lake Natron, contrary to what its name would suggest^17^. Other evaporites that are found at Lake Natron are halite (NaCl) and villiaumite (NaF). Halite does not exhibit a strong spectral variation in the atmospheric window. A reference spectrum of villiaumite was unfortunately not found. Therefore, with the available information, we assign the observed emissivity features to trona, thermonatrite and kogarkoite.

To assess where, how often and with what intensity emissivity features occur, Brightness Temperature Differences (BTDs) can be used. These are differences between affected and unaffected IASI channels and quantitatively express the magnitude of spectral features. A BTD was constructed for both features (the double feature between 830 and 890 cm^−1^ was characterized by a single BTD). Details are shown in Fig. 6. The resulting 2008–2017 averages are shown as insets. The largest values are observed in about the same location as the NH_3_ hotspot, where the lake is exposed most. However, unlike the NH_3_ enhancements, they are also observed over the entire lake surface. The seasonality (see Fig. S3 in the Supplementary Information) reveals that the emissivity features are observed with varying intensity, but throughout the year. Especially the feature associated with the ${{\rm{SO}}}_{4}^{2-}$ group has a clear maximum in December to February, when the lake undergoes rapid cycles of rewetting and evaporation (see Fig. 5). This timing suggests that the observed surface emissivity effects are most pronounced after fresh crystallization. This may also explain why the features are best seen on IASI’s evening overpass (the spectrum of Fig. 6 was observed in the evening). Importantly, note that the seasonality does not match the observed NH_3_ cycle. This largely excludes the possibility of retrieval artefacts in the NH_3_ data related to these significant emissivity features. As a side remark, the above analysis indicates that IASI and other hyperspectral polar orbiters could be exploited for the imaging of surface minerals, something that has been traditionally preserved for imaging instruments^58^.

**Figure 7:**
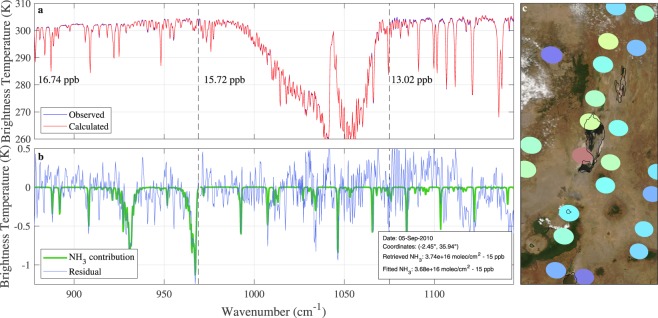
(Panel (a)) Example of an IASI spectrum (blue) where a large NH_3_ column loading is measured and the fitted spectrum (red). Panel (b) shows the residual including the NH_3_ contribution (blue) and the NH_3_ contribution itself (green), see details in the text. MODIS imagery recorded on the same day is shown in panel (c), superimposed are the IASI NH_3_ observations of that day. These are coloured according to the colorbar of Fig. 1 (from 0 to 2 · 10^16^ molec/cm^2^). The ellipses approximate the actual footprint on ground of the measurements. The spectrum on the left corresponds to the red ellipse observed directly over Lake Natron. MODIS imagery is the corrected reflectance imagery from NASA Worldview.

For retrievals that rely on a physical reconstruction of the spectrum, (smaller) retrieval artefacts can be exposed by analysing the difference between the observed and calculated spectrum (i.e. the residual of the fit). However, such residuals are not available as part of the IASI NH_3_ dataset used in this study, as the retrieval algorithm does not attempt to reconstruct the observed spectra. Therefore, to exclude the existence of smaller retrieval artefacts and more importantly to provide explicit spectroscopic evidence of the presence of NH_3_, we performed spectral fits on selected spectra observed over Lake Natron. Figure 7 shows a fit (in red) of a spectrum observed (in blue) on 5 September 2010 over the hotspot area. NH_3_, surface temperature, O_3_ and H_2_O atmospheric concentrations were adjusted to match the observed spectrum via an optimal estimation approach^41^. The retrieval range was limited to 878–1144 cm^−1^ to avoid the sharpest of the emissivity features. Observe that the spectrum is reconstructed adequately over this entire spectral range. From the retrieved parameters, a second spectrum can be simulated that represents what would be observed if NH_3_ was not present in the atmosphere. The difference between the two simulations, with and without NH_3_ in the simulation, allows visualization of the NH_3_ contribution in the fitted spectrum (here shown in green). The difference between the observed spectrum and the simulation without NH_3_, shown here in blue, demonstrates that the NH_3_ signature exceeds the instrumental noise and other features not accounted for in the retrieval. This constitutes the first piece of explicit evidence for the presence of NH_3_.

The fit, presented here, was obtained from three separate retrievals conducted in different spectral bands (878–969 cm^−1^, 970–1075 cm^−1^ and 1074–1144 cm^−1^). This allows obtaining several independent estimates of the NH_3_ column loading from a single spectrum. Surface concentrations of 17, 16 and 13 ppb were obtained for this particular spectrum (IASI measures column loadings, we converted them into estimated surface concentrations by assuming an average vertical profile^59^). Because all three values agree well, the possibility of a retrieval artefact can be ruled out almost entirely. The fact that these results also agree with the 15 ppb value retrieved by the main IASI NH_3_ algorithm provides further confidence on the robustness of the fits, and on the concentrations retrieved by the main algorithm.

Even though the presence of NH_3_ over Lake Natron is confirmed on the 5 September 2010, it is possible that the observed NH_3_ on that day was emitted elsewhere. For this reason, the analysis presented above was repeated on a series of spectra from observations of IASI on Metop A and Metop B. Selected examples are shown in Figs S4 to S7 of the Supplementary Information. To minimize the possibility of observing transported NH_3_, cases were selected where a sharp local NH_3_ enhancement was observed over the hotspot area. Unsurprisingly, all these chosen examples ended up being from September and October. Each of these examples reveal an unambiguous NH_3_ signature with consistent retrieval results across the different bands, providing further confidence that the NH_3_ hotspot is genuine.

## Sources and mechanisms

Due to the absence of outflow, endorheic lakes accumulate in addition to salts also nutrients and organic matter, which maintain via internal cycling the active biological production^60,61^. Decomposition and associated ammonification of nutrient rich organic matter^62,63^ appears to be the principal source of NH_3_ in African soda lakes^22,28,64,65^. This includes breakdown of plankton^66^, droppings of flamingos and other waterbirds^65,67,68^ and miscellaneous organic material carried in via the rivers or hot springs (plant residues, agricultural run-off and waste of mammals and humans^69^). Wetland soil sediments in particular act as a major reservoir of nitrogen and release significant amounts of NH_3_^62,70^, and this is probably not different at Lake Natron whose mudflats are composed of layers of silt and organic material^71^. Flamingos play an important role in the nitrogen cycle of soda lakes, via their feeding and excreting, but also through sediment bioturbation^65,72,73^. However, their precise contribution to the NH_3_ emissions at Lake Natron can only be elucidated with the help of dedicated longterm monitoring data. In terms of input of reactive nitrogen, we expect the feeding and excretion processes of flamingos to average out. The dominant net input to this lake is likely the influx via rivers.

Water measurements in African soda lakes typically yield high concentrations of dissolved organic nitrogen. Reported inorganic nitrogen concentrations are variable^61,64,66^. At Lake Natron, 8 *μ*g/L ${{\rm{NO}}}_{3}^{-}$ and 58 *μ*g/L NH_3_/${{\rm{NH}}}_{4}^{+}$ were reported recently^22^. One probable cause for low NH_3_ concentrations is that the conditions in soda lakes favour volatilization^64,74^. The main argument is that increasing alkalinity and temperature shift the ${{\rm{NH}}}_{4}^{+}$ + OH^−^ $\rightleftharpoons$ NH_3_ + H_2_O equilibrium in a soil or water solution towards the right^75^. As an example, neutral solutions at room temperature contain as much as 99.5% ${{\rm{NH}}}_{4}^{+}$, while for typical Natron conditions of a pH of 10 and a temperature of 35 °C, 92% of ammoniacal N in solution is present as NH_3_^76^ (but see ref.^77^). NH_3_ losses are further promoted by elevated temperatures that favour NH_3_ gas removal at the surface-air interface and strong surface winds over the rift valley floor, which dominate in the dry season^71^.

The cited sources of NH_3_ and these favourable conditions for volatilization alone do not explain the hotspot at Lake Natron, as these elements are also found in other African soda lakes that do not exhibit elevated atmospheric NH_3_ column loadings. A good example of this is Lake Nakuru (see Fig. 1), which is also a soda lake (pH above 10^66,78^), with a much larger anthropogenic input of nitrogen^79^, and with high flamingo populations^24^. The periodic flooding and drying of vast mudflats is however more specific to Lake Natron (other examples are discussed below). Also the timeseries analysis presented before, suggests that the mechanism of massive NH_3_ volatilization should specifically be linked to its drying process. Here we provide six additional processes that may contribute:

1. Evaporative concentration. NH_3_ concentration, is by definition reversely proportional to the amount of liquid. As the soil–water surface dries up, the available NH_3_ concentrates, which increases the surface-atmosphere concentration gradient, resulting in increased volatilization to maintain the equilibrium^40,80^.
2. Convection. Upon soil evaporation, dissolved NH_3_ can be transported to the surface along with the upward movement of water^75^.
3. Decay of plankton. Reduced water availability and increased salinity eventually leads to die-off of plankton and subsequent breakdown and ammonification of the biomass.
4. Assimilation. Reduced biological activity also leads to a decreased uptake of NH_3_.
5. Nitrification. Ammonia oxidizing bacteria can limit nitrogen losses in soda lakes, however this mechanism is no longer available in concentrated brines as nitrification is inhibited beyond a certain salinity threshold^74,81^.
6. Cation exchange and ion pairing. The cation exchange complex determines how much ${{\rm{NH}}}_{4}^{+}$ can be reversible adsorbed on soil colloids. Silts and organic material in principle favour a high cation exchange coefficient and therefore help retaining ${{\rm{NH}}}_{4}^{+}$ in the soil^82^. However, high concentrations of Na^+^ can outcompete and displace ${{\rm{NH}}}_{4}^{+}$ in the available soil exchange sites. In addition, ion pair formation with anions in the surface water may stimulate diffusion of ${{\rm{NH}}}_{4}^{+}$ out of the sediment^81,83,84^.

**Figure 8:**
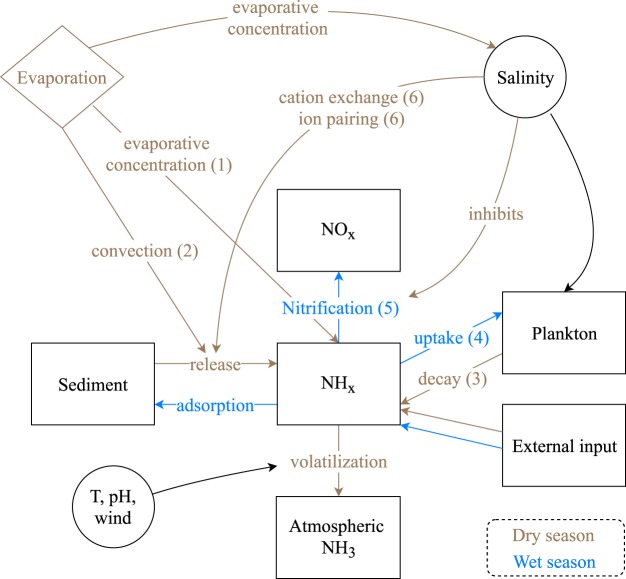
A simplified conceptual model of the different processes that link evaporation and soil drying to NH_3_ volatilization. The numbers in parentheses refer to the mechanisms identified in Sec. 5.

A simplified conceptual model of the above processes is presented in Fig. 8. The positive correlation between salinity and NH_3_ concentration, expected from several of these, has been reported by *in*-*situ* measurements in both saline^81^ and soda lakes^65,85^.

We consider briefly the question whether elevated NH_3_ column loadings may be observed over other nearby Eastern Rift Valley lakes. At least four soda lakes are shallow and have regularly flooded/exposed saline mudflats (see Fig. 1): Lake Eyasi, Lake Manyara, Lake Magadi and Lake Logipi. These are also important flamingo lakes^86^. Lake Magadi neighbours Lake Natron and resembles it in many ways. However, its mudflats are much smaller in surface area which may explain why no convincing enhancements are observed. A small NH_3_ hotspot is detected at Lake Logipi^61^, over mudflats just south of the permanent water body. The location and timing are consistent with the patterns observed at Lake Natron, but relatively low column loadings, larger year-to-year variability and the absence of a clear seasonal cycle make it even more challenging to analyse. Enhancements over Lake Logipi are seen in the years 2008 to 2012 (2012 is the record year). Surprisingly, in subsequent years 2013–2016 the hotspot disappeared, but re-emerged in 2017. A small hotspot is seen near the centre of Lake Manyara, but a larger background makes it difficult to conclude anything. No NH_3_ hotspot is seen at Lake Eyasi.

It is our hope that the results presented in this study will spur on future studies driven by *in situ* measurements of nutrient concentrations, nitrogen dynamics and observations of environmental factors at Lake Natron and other similar lakes. Examples of the latter would be data on biomass growth and die-off, and on the distribution of the highly nomadic lesser flamingos. Especially temporal data that can be linked with the timeseries presented here would be most welcome (e.g. the few bird censuses that are available date before 2007^87–90^, or are for other lakes^72,91^). Such ground-truth data will allow determining the dominant mechanisms at play and ultimately improve our understanding of the nitrogen cycle in soda lakes.

## Supplementary information

Supplementary info

## Data Availability

The IASI data used in this study has been archived in the PANGAEA repository (10.1594/PANGAEA.895632); Other IASI NH_3_ data is available from the Aeris data infrastructure (http://iasi.aeris-data.fr).
