# Supplementary material for: Atmospheric ammonia (NH3) emanations from Lake Natron’s saline mudflats
Source: Sci Rep. 2019 Mar 14;9:4441. doi: 10.1038/s41598-019-39935-3 (PMC6418304; doi:10.1038/s41598-019-39935-3)
Supplement: Supplementary file 1 — Supplementary info [file 41598_2019_39935_MOESM1_ESM.pdf]

Supplementary information

Atmospheric ammonia (NH_3) emanations from Lake Natron's saline mudflats

L. Clarisse[1,*], M. Van Damme[1], W. Gardner[2], P.-F. Coheur[1], C. Clerbaux[3,1], S. Whitburn[1], J. Hadji-Lazaro[3], and D. Hurtmans[1]

[1]Université libre de Bruxelles (ULB), Atmospheric Spectroscopy, Service de Chimie Quantique et Photophysique, Brussels, Belgium
[2]The University of Texas at Austin, Marine Science Institute, 750 Channel View Drive, Port Aransas, Texas 78373, United States
[3]LATMOS/IPSL, Sorbonne Universités, UVSQ, CNRS, Paris, France
[*]lclariss@ulb.ac.be

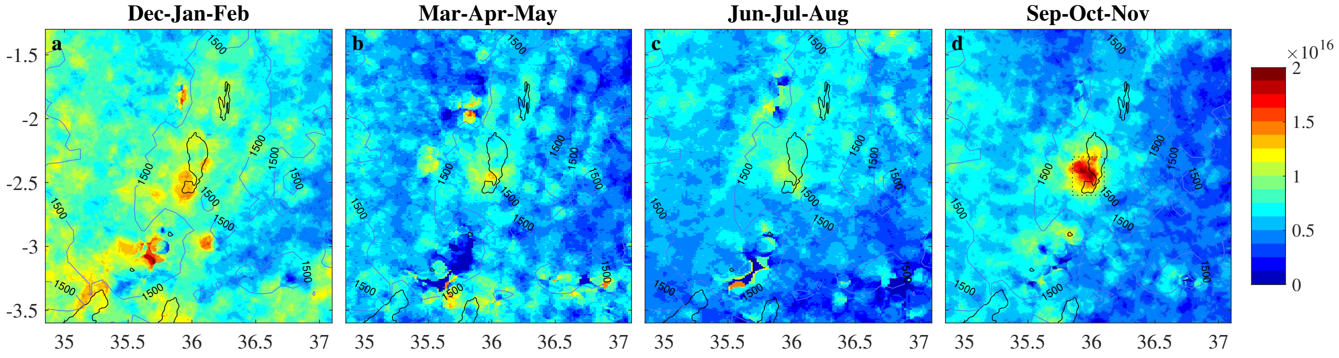

Figure S1. Same as Fig. 3 but for IASI onboard Metop B and for the timeperiod 2013–2017.

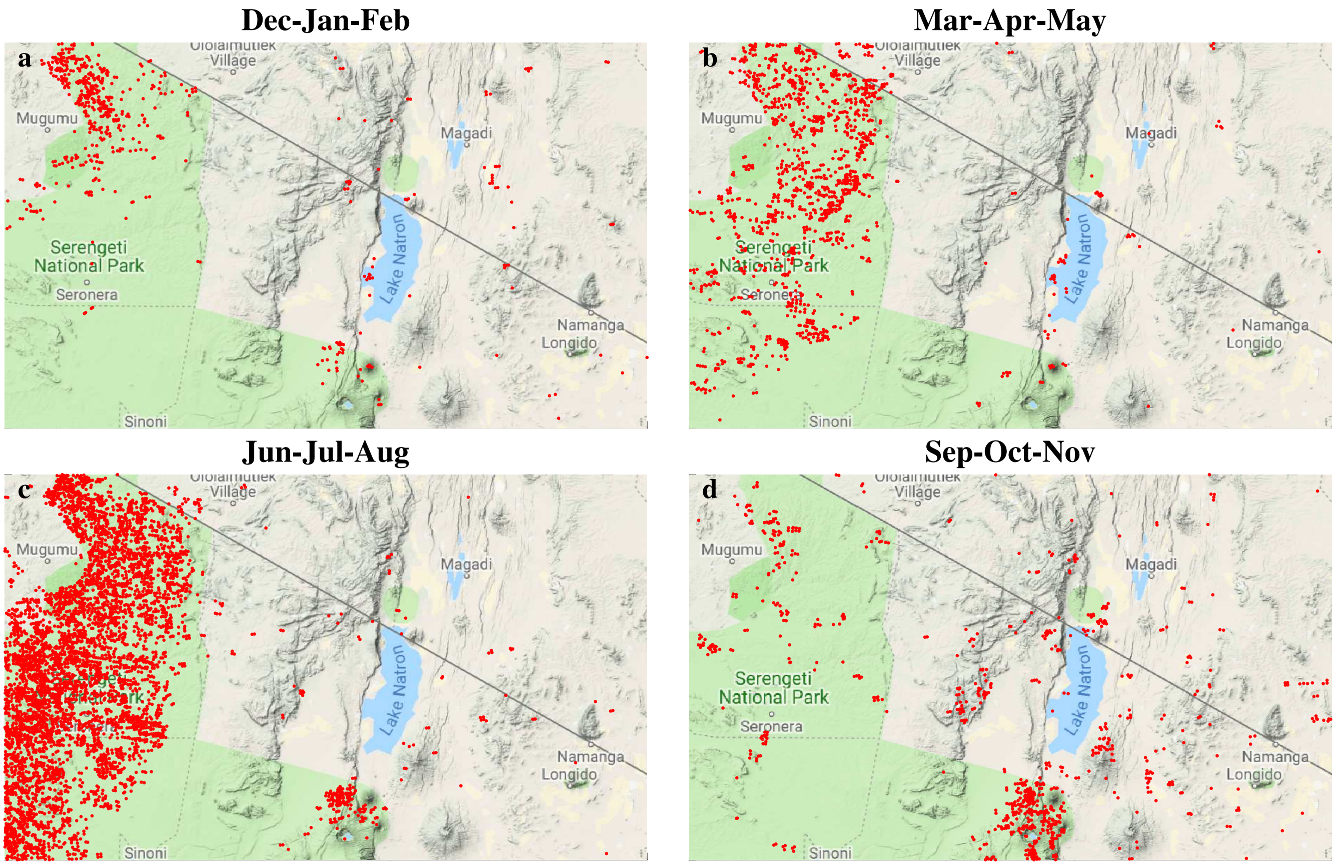

Figure S2. Seasonal MODIS detected fires (red dots) for the period 2008-2017. Map data: Google Maps.

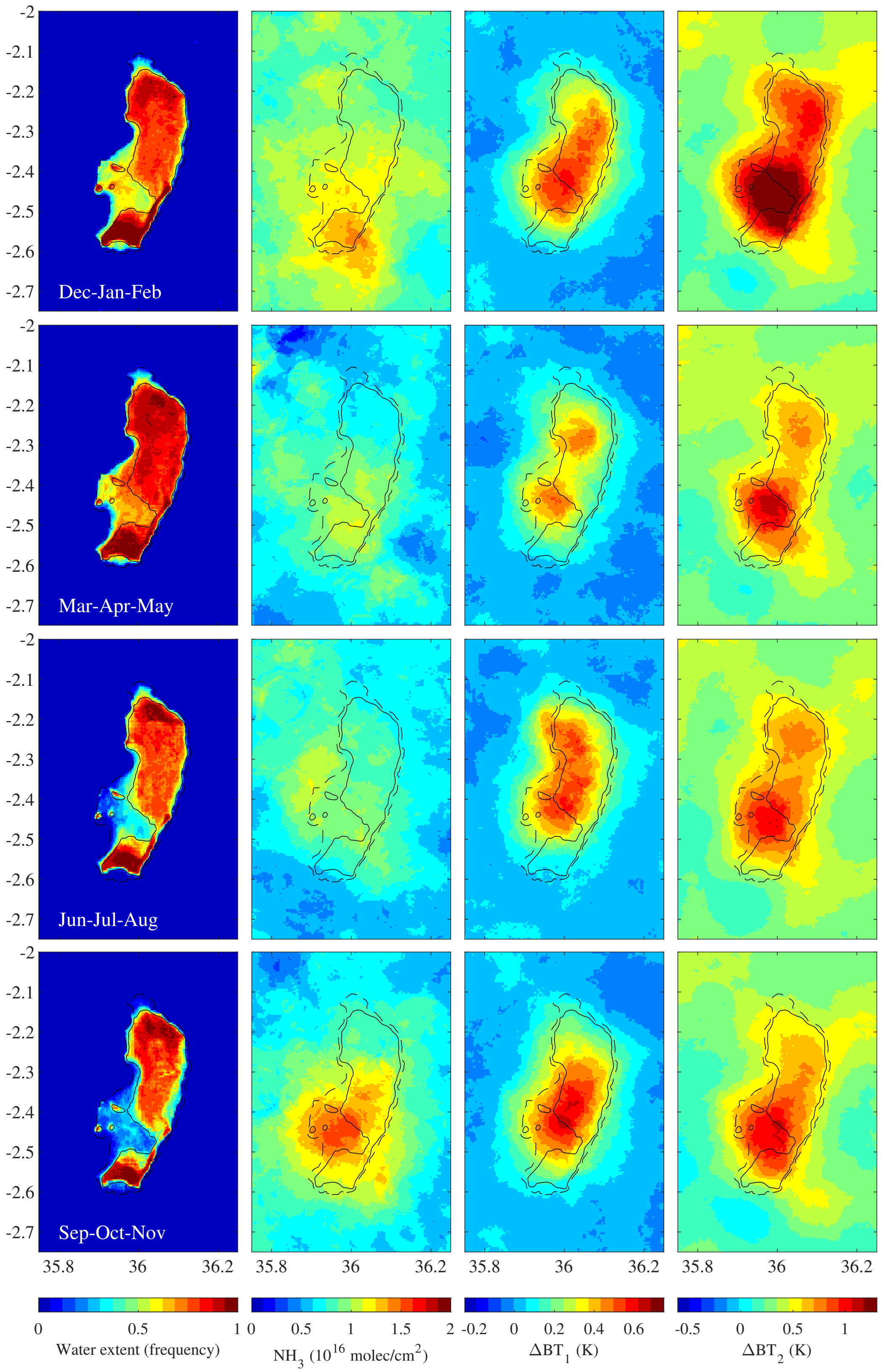

Figure S3. Seasonal averages of the water extent (2013–2017) derived from MODIS and of NH_3 column loadings and brightness temperature differences (2008–2017) from IASI data. Δ BT_1 and Δ BT_2 refer to the brightness temperature differences defined in Fig. 6.

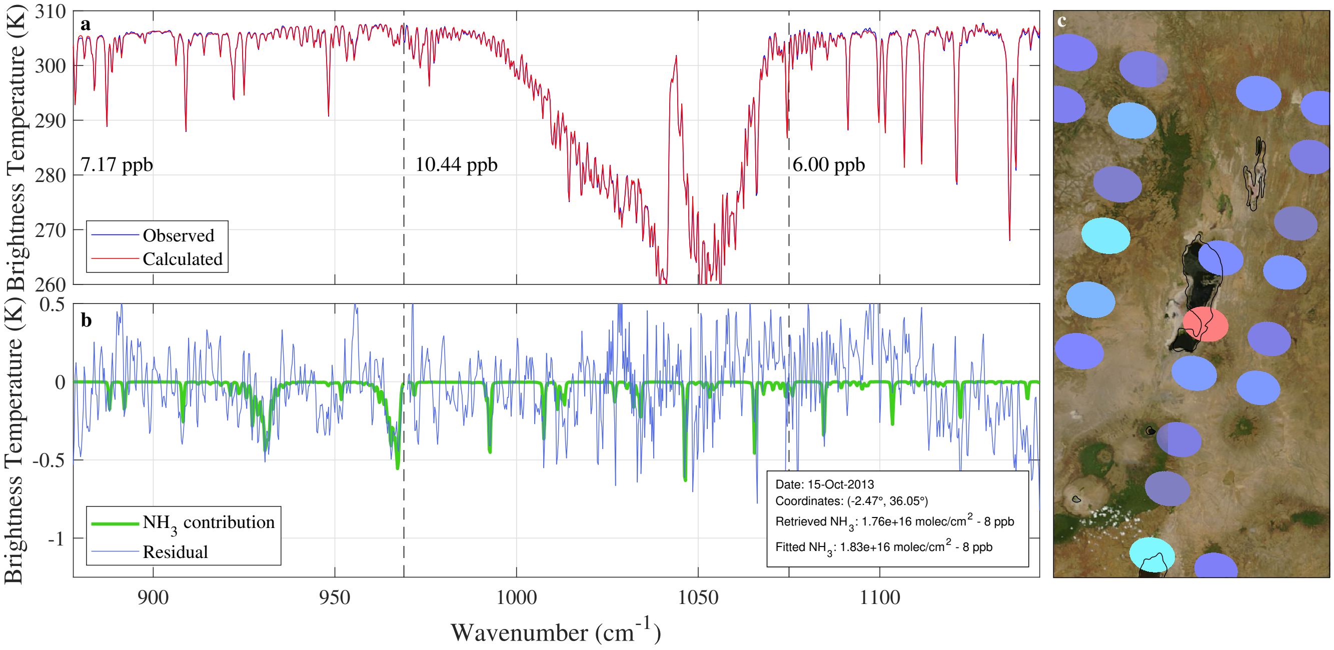

Figure S4. Same as Fig. 7, but for 15 October 2013 (Metop A). MODIS imagery is the corrected reflectance imagery from NASA Worldview.

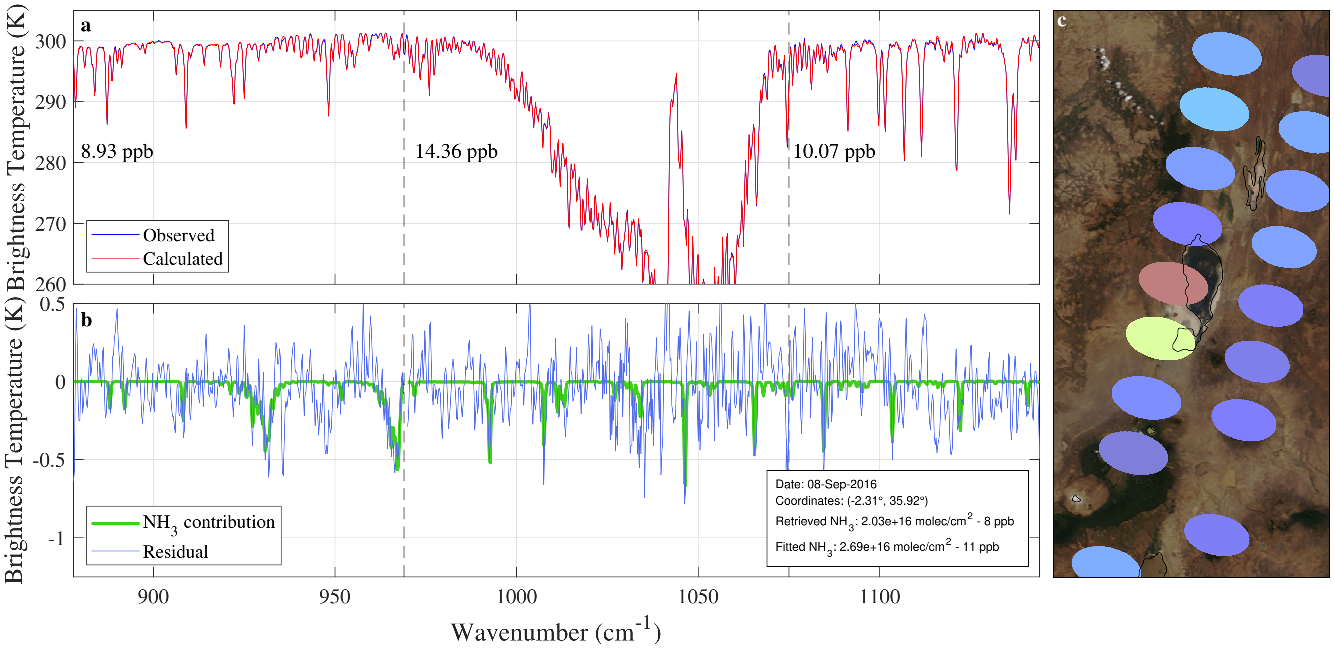

Figure S5. Same as Fig. 7, but for 8 September 2016 (Metop A). MODIS imagery is the corrected reflectance imagery from NASA Worldview.

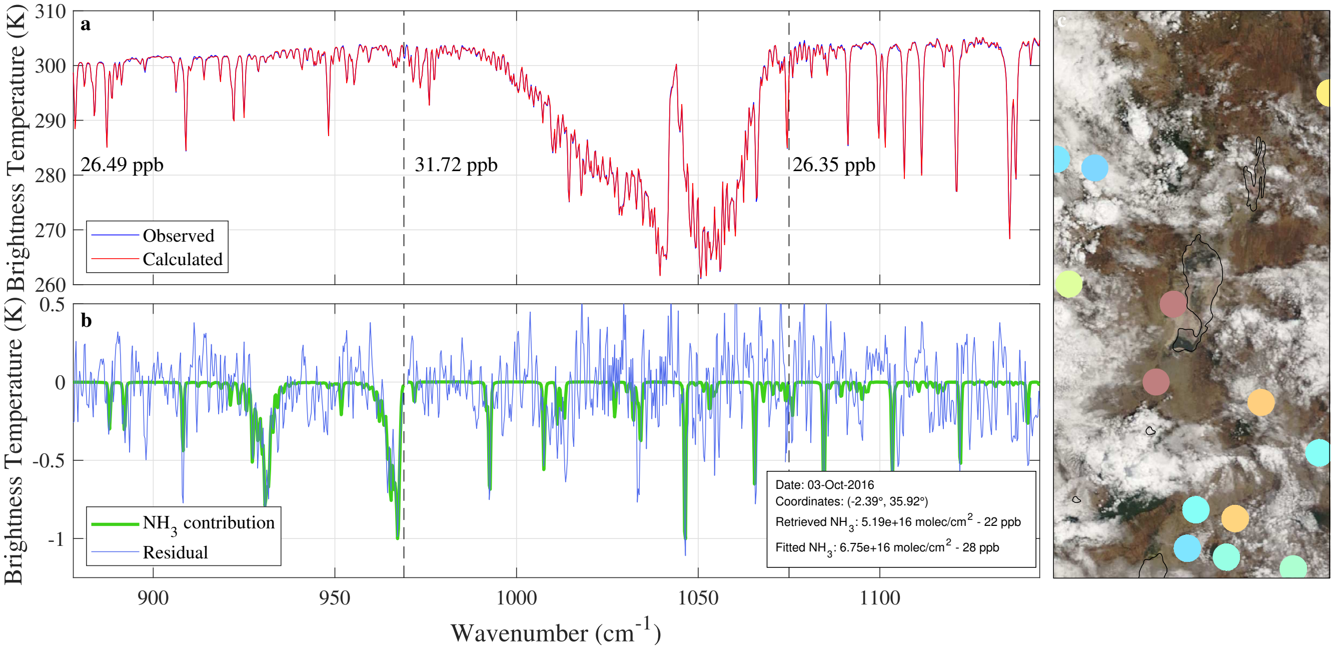

Figure S6. Same as Fig. 7, but for 3 October 2016 (Metop B). MODIS imagery is the corrected reflectance imagery from NASA Worldview.

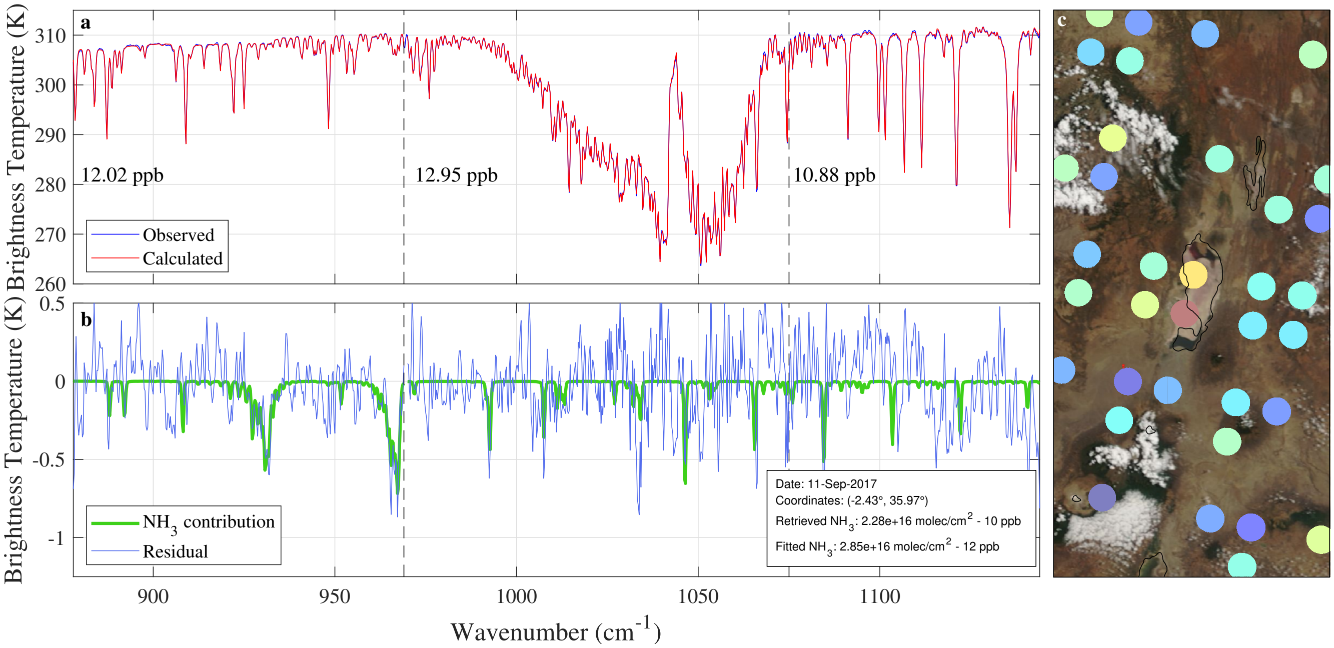

Figure S7. Same as Fig. 7, but for 11 September 2017 (Metop B). MODIS imagery is the corrected reflectance imagery from NASA Worldview.
